# Cryopreservation of two species of the multicellular volvocine green algal genus *Astrephomene*

**DOI:** 10.1186/s12866-023-02767-3

**Published:** 2023-01-18

**Authors:** Hisayoshi Nozaki, Fumi Mori, Yoko Tanaka, Ryo Matsuzaki, Shota Yamashita, Haruyo Yamaguchi, Masanobu Kawachi

**Affiliations:** 1 Biodiversity Division, National Institute for Environmental Studies, Tsukuba, Ibaraki 305-8506 Japan; 2 Department of Biological Sciences, Graduate School of Science, The University of Tokyo, Bunkyo-ku, Tokyo, 113-0033 Japan; 3 Present Address: Department of Gene Function and Phenomics, National Institute of Genetics, 1111 Yata, Mishima, Shizuoka 411-8540 Japan

**Keywords:** Asexual cycle, Astrephomene, Cryopreservation, Cryoprotectant, Culture collection, Volvocine green algae

## Abstract

**Background:**

*Astrephomene* is an interesting green algal genus that, together with *Volvox*, shows convergent evolution of spheroidal multicellular bodies with somatic cells of the colonial or multicellular volvocine lineage. A recent whole-genome analysis of *A. gubernaculifera* resolved the molecular-genetic basis of such convergent evolution, and two species of *Astrephomene* were described. However, maintenance of culture strains of *Astrephomene* requires rapid inoculation of living cultures, and cryopreserved culture strains have not been established in public culture collections.

**Results:**

To establish cryopreserved culture strains of two species of *Astrephomene*, conditions for cryopreservation of the two species were investigated using immature and mature vegetative colonies and two cryoprotectants: N,N-dimethylformamide (DMF) and hydroxyacetone (HA). Rates of cell survival of the *A. gubernaculifera* or *A. perforata* strain after two-step cooling and freezing in liquid nitrogen were compared between different concentrations (3 and 6%) of DMF and HA and two types of colonies: immature colonies (small colonies newly released from the parent) and mature colonies (large colonies just before daughter colony formation). The highest rate of survival [11 ± 13% (0.36–33%) by the most probable number (MPN) method] of *A. gubernaculifera* strain NIES-4017 (established in 2014) was obtained when culture samples of immature colonies were subjected to cryogenic treatment with 6% DMF. In contrast, culture samples of mature colonies subjected to 3% HA cryogenic treatment showed the highest “MPN survival” [5.5 ± 5.9% (0.12–12%)] in *A. perforata*. Using the optimized cryopreservation conditions for each species, survival after freezing in liquid nitrogen was examined for six other strains of *A. gubernaculifera* (established from 1962 to 1981) and another *A. perforata* strain maintained in the Microbial Culture Collection at the National Institute for Environmental Studies (MCC-NIES). We obtained ≥0.1% MPN survival of the *A. perforata* strain. However, only two of the six strains of *A. gubernaculifera* showed ≥0.1% MPN survival. By using the optimal cryopreserved conditions obtained for each species, five cryopreserved strains of two species of *Astrephomene* were established and deposited in the MCC-NIES.

**Conclusions:**

The optimal cryopreservation conditions differed between the two species of *Astrephomene*. Cryopreservation of long-term-maintained strains of *A. gubernaculifera* may be difficult; further studies of cryopreservation of these strains are needed.

**Supplementary Information:**

The online version contains supplementary material available at 10.1186/s12866-023-02767-3.

## Background

**Fig. 1:**
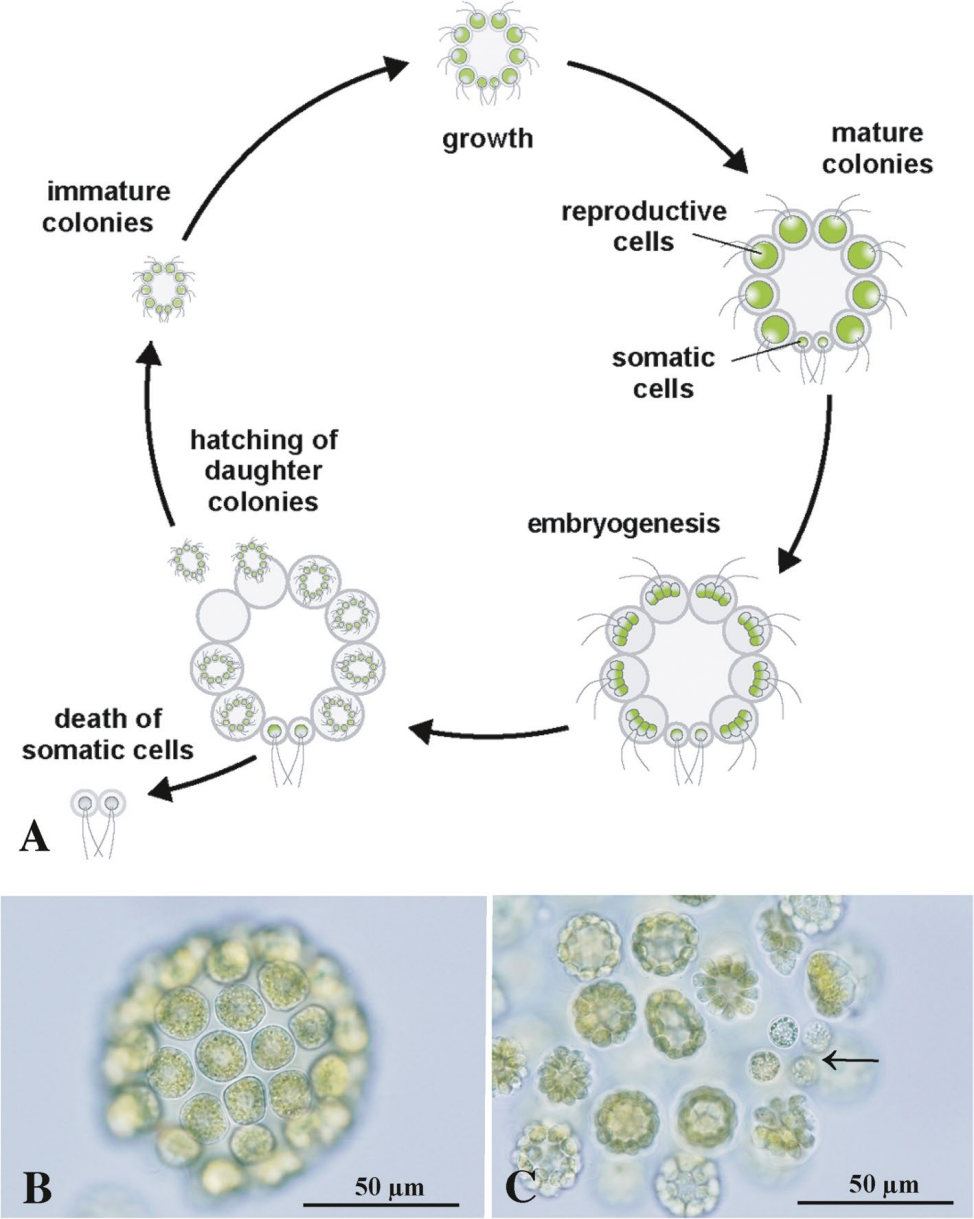
Asexual life cycle of *Astrephomene*. **A** Diagram of asexual life cycle of *Astrephomene*. Based on Nozaki [9] and Yamashita et al. [10]. Colonies are generally 32- or 64-celled with two or four somatic cells in the posterior pole. Each reproductive cell performs cell divisions to produce a daughter colony. **B** Mature vegetative colony of *A. gubernaculifera* strain NIES-4017. **C** Posterior portion of parental colony of *A. gubernaculifera* strain NIES-4017 showing embryogenesis of reproductive cells and four undivided somatic cells (arrow)

The volvocine green algae are composed of the unicellular genus *Chlamydomonas* and multicellular genera such as *Gonium* and *Volvox* (Additional file 1: Fig. S1). Because these green algae represent a unique model lineage for experimental studies of the evolution of sex and multicellularity [1], multicellular volvocine algae have been investigated in molecular and genomics studies [2–4]. Among the volvocine green algae, two independent lineages, Volvocaceae (including *Volvox*) and *Astrephomene* (Fig. 1), show similar or convergent evolution of multicellular spheroidal bodies with germ-soma differentiation (Additional file 1: Fig. S1) [5–7]. Whole-genome sequencing of *A. gubernaculifera* provided insight into the molecular-genetic basis of such convergent evolution [8]. Thus, *Astrephomene* represents a hopeful key organism for studies of multicellularity and germ-soma differentiation.

The genus *Astrephomene* was originally described by Pocock [11] based on a single species: *A. gubernaculifera*. Using culture strains of *A. gubernaculifera* originating from the USA and Mexico, morphology, sexual isolation within the morphological species, and physiology were studied [12–14]. The second species, *A. perforata*, was described based on clonal cultured materials from Japan [9]. *A. perforata* differs from *A. gubernaculifera* in the morphology of the individual sheaths of cells in the spheroid and pyrenoids in the chloroplast [9]. Fifteen strains of the two species of *Astrephomene* established in these studies were deposited in the Culture Collection of Algae at the University of Texas at Austin (CCA-UTEX) [15]. Nine strains of the two *Astrephomene* species are available from the MCC-NIES (https://mcc.nies.go.jp/index_en.html [16]) and one strain of *A. gubernaculifera* is available from the Culture Collection of Algae at Goettingen University (SAG) (https://uni-goettingen.de/en/45175.html [17]). Since these culture strains are maintained by serial inoculations of living cells to new media, high costs are carried in the culture collections. However, cryopreserved culture strains of *Astrephomene* have not been established.

Although Mori et al. [18] examined cell survival after freezing in liquid nitrogen in six strains of two species of *Astrephomene* maintained in the MCC-NIES [16] (https://mcc.nies.go.jp/index_en.html) by using dimethyl sulfoxide (DMSO) as a cryoprotectant, none survived freezing. Later, Nakazawa & Nishii [19] demonstrated poor recovery (i.e., recovery of one or two of three replicates) of *A. gubernaculifera* strain NIES-418 after cryopreservation in liquid nitrogen when N,N-dimethylformamide (DMF) or hydroxyacetone (HA) was used as a cryoprotectant for two-step freezing. However, no other studies of the cryopreservation or establishment of cryopreserved strains of *Astrephomene* have been performed.

This study was undertaken to determine the optimal conditions for cryopreservation of culture strains of two species of *Astrephomene*. Optimal conditions for the cryopreservation of the two species were determined using immature and mature colonies from the asexual cycle of the two *Astrephomen*e species (Fig. 1) and two cryoprotectants (DMF and HA). By using these conditions, cryopreserved strains of the two species were established.

## Materials and methods

### Culture strains

Nine culture strains of two species of *Astrephomene* maintained at the MCC-NIES [16] were used (Table 1). The cultures were grown in screw-cap tubes (18 × 150 mm) containing 10 mL of *Volvox* thiamin acetate (VTAC) medium or urea soil *Volvox* thiamin (USVT) medium [16] at 25 °C, with a 12 h:12 h light:dark schedule under cool-white fluorescent lamps at an intensity of 100–130 μmol m^− 2^ s^− 1^. To maintain the cultures, USVT medium was used for *A. gubernaculifera* strain NIES-853, whereas the other six strains of *A. gubernaculifera* and two strains of *A. perforata* were cultured in VTAC medium.

**Table 1:** List of strains of two species of *Astrephomene* used in this study

Species	Strain designation	Locality (Date of collection)^a^	Date of establishment of strain^a^	Growth medium
*A. gubernaculifera*	NIES-4017	Chiba, Japan (July 2014)	2014 October	VTAC
	NIES-418	Kanagawa, Japan (April 1981)	1981 May	VTAC
	NIES-419	Kanagawa, Japan (April 1981)	1981 May	VTAC
	NIES-628	Kanagawa, Japan (December 1980)	1981 July	VTAC
	NIES-853 (UTEX 1392)	Michigan, USA (July 1961)	1962 June	USVT
	NIES-854 (UTEX 1394)	Indiana, USA (October 1962)	1962 October	VTAC
	NIES-855 (UTEX 1398)	California, USA (August 1953)	1965 April	VTAC
*A. perforata*	NIES-564 (UTEX 2474)	Kanagawa, Japan (December 1980)	1981 June	VTAC
	NIES-565 (UTEX 2475)	Kanagawa, Japan (December 1980)	1981 June	VTAC

^a^From Nozaki [9], Starr & Zeikus [15], Kawachi et al. [16] and Yamashita et al. [10]

**Fig. 2:**
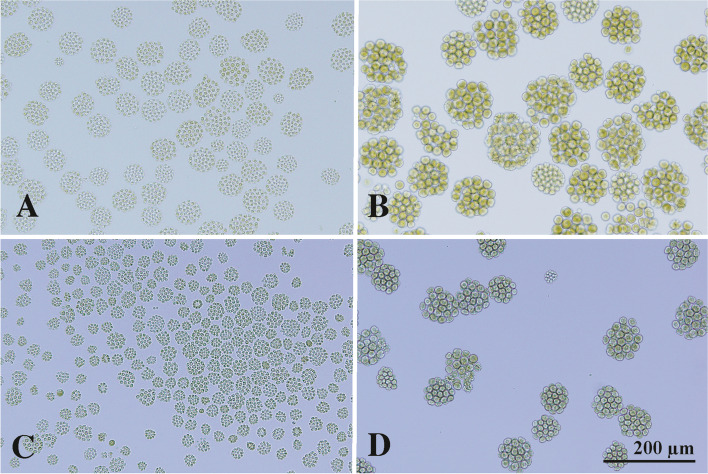
Immature and mature colonies of two species of *Astrephomene* (Fig. 1A) that were used for cryogenic treatments (Table 2), shown at the same magnification throughout. **A** Immature colonies of *A. gubernaculifera* strain NIES-4017. **B** Mature colonies of *A. gubernaculifera* strain NIES-4017. **C** Immature colonies of *A. perforata* strain NIES-564. **D** Mature colonies of *A. perforata* strain NIES-564

To prepare cultures of immature colonies (newly released small daughter colonies with reproductive cells approximately 5 μm in diameter) (Fig. 2A, C), 0.2–0.3 mL of 4–5-day-old cultures (approximately 10^6^ cells/mL) were inoculated into 10 mL of USVT medium in a screw-cap tube 4–6 h after the onset of the light period of the 12 h:12 h light:dark cycle. The inoculated cultures were incubated for 48 h at 25 °C with a 12 h:12 h light:dark cycle, as described above. Cultures of mature colonies (large colonies just before daughter colony formation, with reproductive cells approximately 15 μm in diameter) (Fig. 2B, D) were obtained as described above, except that the inoculum was diluted 30–50-fold with USVT medium.

### Cryopreservation

The optimal cryopreservation conditions for two species of *Astrephomene* were assessed using DMF or HA as a cryoprotectant. Nakazawa and Nishii [19] demonstrated partial survival of *A. gubernaculifera* cells after freezing in liquid nitrogen with 3% DMF and 3% HA. Nakazawa and Nishii [19] studied the cryopreservation of multicellular volvocine algae using 0.25 mL PCR tubes as vials for two-step freezing. However, we recently demonstrated that use of 2 mL cryotubes (Cryo.s, 2 mL, Round Bottom, Starfoot Base; Greiner Bio-One, Kremsmünster, Austria) as vials resulted in a higher survival rate than achieved using 0.20 mL PCR tubes for cryopreservation of the multicellular volvocine alga *Gonium pectorale* [20]. Thus, we prepared 1.0 mL samples in 2 mL cryotubes for cryopreservation of two species of *Astrephomene*, and eight cryopreservation conditions were examined for *A. gubernaculifera* strain NIES-4017 and *A. perforata* strain NIES-564: immature colonies in 3% DMF, immature colonies in 6% DMF, immature colonies in 3% HA, immature colonies in 6% HA, mature colonies in 3% DMF, mature colonies in 6% DMF, mature colonies in 3% HA, and mature colonies in 6% HA. For cryopreservation, a 48-day-old culture of immature or mature colonies (see above) actively growing in USVT medium (2–4 mL) was mixed with an equal volume of USVT medium containing 6% or 12% DMF (or HA) to prepare a sample with 3% or 6% DMF (or HA), respectively. The cells were exposed to the cryoprotectant at room temperature (20–25 °C) for 15 min. Next, 1.0 mL of the culture sample with DMF (or HA) was transferred to a 2 mL cryotube. The sample cryotube was subjected to two-step cooling in liquid nitrogen [18, 20, 21]. Cell suspensions in tubes were frozen in vapor-phase liquid nitrogen at a rate of − 1 °C/min to − 40 °C using a programmable freezer (Controlled Rate Freezer, KRYO 560-16; Planer, Sunbury-on-Thames, UK). After 15 min of maintenance at − 40 °C, the cell suspensions were cooled rapidly to − 196 °C by immersion in liquid nitrogen, and finally stored at − 190 °C in vapor-phase liquid nitrogen. To assess the viability of cells frozen in liquid nitrogen, the frozen samples in tubes were thawed in a 40 °C or 60 °C water bath while the tube was shaken by hand until the ice crystals almost disappeared (approximately 120 or 75 s, respectively); then, 0.1 mL of the diluted sample was immediately subjected to analysis using the most probable number (MPN) method [18, 20–23]. For the MPN method, eight wells in each dilution series of a 48-well microplate (CellStar Cell Culture Multiwell Plate with Lid, Greiner Bio-One) were filled with 0.9 mL of USVT medium. Three replicates of eight 1/10th dilutions were performed for each cryotube of sample using a 6-channel pipette (Pipet-Lite Adjustable Spacer LA6-1200XLS; Mettler-Toledo, Greifensee, Switzerland). As a control, three replicates of eight 1/10th dilutions of cultures without cryogenic treatment and cryoprotectant were treated in the same manner. The plates were initially incubated in darkness at 25 °C for 2 days, then transferred to a 12 h:12 h light:dark schedule at 25 °C for 2 weeks [20]. Each well was scored for growth and MPN values (cell numbers) were estimated based on those scores using MPN Calculator 3.1 (https://softdeluxe.com/MPN-Calculator-444229/) [24, 25]. The recovery rate of viable cells (%) was calculated relative to the viable cell count in the unfrozen control using the MPN method. For each of the four types of cryopreservation conditions, recovery rates were measured based on six tubes from two independent experiments (Table 2).

**Table 2:** Comparison of results of eight types of cryopreservation conditions for two species of *Astrephomene* based on most probable number (MPN) methods

Conditions for cryopreservation (species)	Total viability^a^ (range) %	Experiment I	Experiment II
		MPN cell numbers in three cryotubes (/mL) (control); [Number of viable cultures by 1st (2nd) inoculation with 3 (3) 10 mL cultures]	MPN cell numbers in three cryotubes (/mL) (control); [Number of viable cultures by 1st (2nd) inoculation with 3 (3) 10 mL cultures]
Immature colonies in 3% DMF (AG^b^)	0.36 ± 0.38 (0.022-1.0)	92, 92, 230 (22000); [3(2)]	9.2, 23, 92 (42000); [3(0)]
Immature colonies in 6% DMF (AG)	11 ± 13 (0.36-33)	2300, 4200, 7300 (22000); [3(3)]	150, 230, 230 (42000); [3(3)]
Immature colonies in 3% HA (AG)	0.24 ± 0.20 (0.014-0.46)	3, 7.3, 74 (22000); [3(1)]	140, 420, 420 (92000); [3(3)]
Immature colonies in 6% HA (AG)	0 ± 0 (0-0)	0, 0, 0 (22000); [0(0)]	0, 0, 0 (92000); [0(0)]
Mature colonies in 3% DMF (AG)	0 ± 0 (0-0)	0, 0, 0 (22000); [0(0)]	0, 0, 0 (9200); [0(0)]
Mature colonies in 6% DMF (AG)	0 ± 0 (0-0)	0, 0, 0 (22000); [2(0)]	0, 0, 0 (9200); [0(0)]
Mature colonies in 3% HA (AG)	0 ± 0 (0-0)	0, 0, 0 (22000); [2(1)]	0, 0, 0 (14000); [3(0)]
Mature colonies in 6% HA (AG)	0 ± 0 (0-0)	0, 0, 0 (22000); [0(0)]	0, 0, 0 (14000); [0(0)]
Immature colonies in 3% DMF (AP^c^)	0.053 ± 0.098 (0-0.25)	23, 42, 230 (92000); [3(2]]	0, 0, 0 (9200); [0(0)]
Immature colonies in 6% DMF (AP)	0.032 ± 0.037 (0-0.1)	9.2, 9.2, 92 (92000); [3(1)]	0, 3, 3.6 (9200); [3(1)]
Immature colonies in 3% HA (AP)	2.2 ± 1.9 (0.3-4.6)	920, 1500, 4200 (92000); [3(3)]	28, 92, 420 (9200); [3(3)]
Immature colonies in 6% HA (AP)	0.071 ± 0.077 (0-0.14)	0, 0, 0 (92000); [0(0)]	13, 13, 13 (9200); [3(3)]
Mature colonies in 3% DMF (AP)	0 ± 0 (0-0)	0, 0, 0 (7400); [1(0)]	0, 0, 0 (9200); [0(0)]
Mature colonies in 6% DMF (AP)	0.093 ± 0.12 (0-0.31)	9.2, 9.2, 23 (7400); [3(1)]	0, 0, 0 (9200); [3(0)]
Mature colonies in 3% HA (AP)	5.5 ± 5.9 (0.12-12)	740, 740, 920 (7400); [3(3)]	11,15, 21 (9200); [3(3)]
Mature colonies in 6% HA (AP)	0 ± 0 (0-0)	0, 0, 0 (7400); [0(0)]	0, 0, 0 (9200); [0(0)]

^a^Significant difference (*p* < 0.01) was detected in interactions between *Astrephomene* species and cryoprotectants, based on unweighted-mean ANOVA analyzed by js-STAR XR release 1.6.6j <http://www.kisnet.or.jp/nappa/software/star/index.htm>

^b^*A. gubernaculifera* strain NIES-4017

^c^*A*. *perforata* strain NIES-564

In addition, immediately after thawing of the three frozen cryotubes of each sample, 0.5 mL of the melted sample in each cryotube was inoculated into fresh growth medium (10 mL) in a six-well plate (PS with Lid; Greiner Bio-One) (first inoculation); subsequently, 0.5 mL of the first inoculation was transferred to 10 mL of fresh growth medium (second inoculation) in a six-well plate to confirm the recovery of frozen and thawed cells in the same volume of culture medium used in the MCC-NIES.

## Results

“MPN survival” after the eight cryogenic treatments differed between the two species of *Astrephomene* (Table 2). For *A. gubernaculifera* strain NIES-4017, the highest recovery rate after freezing in liquid nitrogen and thawing was achieved when immature colonies were subjected to 6% DMF during two-step freezing (11 ± 13% MPN survival, Table 2). In addition, recovery of active growth was observed in the six 10 mL cultures after two successive inoculations of liquid nitrogen-frozen cultures of immature colonies of *A. gubernaculifera* strain NIES-4017 using 6% DMF (Table 2). However, 0% MPN survival and partial recovery of active growth in six 10 mL cultures after two successive inoculations were observed in samples of mature colonies of *A. gubernaculifera* strain NIES-4017 treated with DMF or HA (Table 2). In contrast, < 0.1% MPN survival was found in samples of *A. perforata* strain NIES-564 treated with 3% HA, with mature or immature colonies (Table 2). The highest rate of MPN survival among the eight cryogenic treatments in *A. perforata* strain NIES-564 was 5.5 ± 5.9% when mature colonies were mixed with 3% HA during two-step freezing (Table 2).

Because the effect of sample parameters (colony maturation and cryoprotectants) on recovery after cryopreservation were species-specific, recovery based on the MPN method and two successive inoculations in 10 mL of new medium after the cryopreservation of seven other strains of *Astrephomene* was examined using immature colonies of *A. gubernaculifera* with 6% DMF, or mature colonies of *A. perforata* with 3% HA. Based on these potentially optimized cryopreserved conditions for each species (Table 2), we obtained ≥0.1% MPN viability rates and active growth based on two successive inoculations in 10 mL cultures of *A. gubernaculifera* strains NIES-418 and NIES-853, and *A. perforata* strain NIES-565 (Table 3). However, the other four strains of *A. gubernaculifera* did not recover after freezing in liquid nitrogen and thawing (≥ 0.1% MPN viability), and did not grow after one and two inoculations to 10 mL of medium (Table 3). Thus, five cryopreserved strains of *Astrephomene* were deposited in the MCC-NIES.

**Table 3:** Comparison of recovery results of seven strains of two *Astrephomene* species (Table 1) after possible optimal cryogenic treatment (immature colonies of *A. gubernaculifera* [AG] with 6% DMF, or mature colonies of *A. perforata* [AP] with 3% HA; Table 2) in liquid nitrogen

Strain designation [species]	Total viability (range) %	Experiment I	Experiment II
		MPN cell numbers in three cryotubes (/mL) (control); [Number of viable cultures by 1st (2nd) inoculation with 3 (3) 10 mL cultures]	MPN cell numbers in three cryotubes (/mL) (control); [Number of viable cultures by 1st (2nd) inoculation with 3 (3) 10 mL cultures]
NIES-418 [AG]	0.570 ± 0.88 (0.0026-2.2)	7.3, 15, 15 (280000); [3(3)]	92, 420, 920 (42000); [3(3)]
NIES-419^a^ [AG]	0 (0-0)	0, 0, 0 (81000); [0(0)]	0, 0, 0 (15000); [0(0)]
NIES-628^a^ [AG]	0.032 ± 0.0020 (0.011-0.065)	9.2, 11, 20 (81000); [3(3)]	9.2, 9.2, 15 (23000); [3(2)]
NIES-853 [AG]	0.54 ± 0.19 (0.41-0.91)	380, 420, 740 (81000); [3(3)]	38, 42, 42 (9200); [3(3)]
NIES-854^a^ [AG]	0 (0-0)	0, 0, 0 (40000); [0(0)]	0, 0, 0 (9200); [1(0)]
NIES-855^a^ [AG]	0 (0-0)	0, 0, 0 (180000); [0(0)]	0, 0, 0 (23000); [0(0)]
NIES-565 [AP]	9.6 ± 5.0 (3.8-18)	1500, 2300, 4200 (40000); 3 [3]	2100, 2300, 4200 (23000); [3(3)]

^a^Not used for cryopreserved strain in MCC-NIES because of < 0.1% MPN survivability

## Discussion

Because *Astrephomene* requires organic compounds such as acetate for photoheterotrophy, and grows extremely rapidly under photoheterotrophic conditions [14] (Additional file 1: Fig. S2; Additional file 2: Text S1), serial inoculations of living cells to new media during short intervals are required for maintenance of living cultures [10, 16] (https://mcc.nies.go.jp/index_en.html). In addition, during the long-term maintenance of growing cultures by subculturing, the ability to perform normal morphogenesis gradually decreases in *Astrephomene* [10]. Thus, cryopreservation of culture strains of *Astrephomene* is needed.

In the present study, we determined the optimal liquid-nitrogen cryopreservation conditions for *A. gubernaculifera* strain NIES-4017 and *A. perforata* strain NIES-564 by selecting mature or immature colonies of *Astrephomene* and two types of cryoprotectants, DMF and HA (Table 2). Amidic and acetonic cryoprotectants, such as DMF and HA, enable cryopreservation of cells based on their ability to cross the cell membrane and cytotoxic effects [19]. We examined MPN survival of unfrozen cells of mature and immature colonies of two species of *Astrephomene* treated with 3% DMF, 6% DMF, 3% HA and 6% HA (Additional file 1: Table S1, Fig. S3). When immature colonies were treated with 6% DMF, unfrozen cells of *A. gubernaculifera* strain NIES-4017 exhibited a moderate survival rate (39%), but frozen NIES-4017 cells showed the highest survival rate (11%) among all frozen cell types. By contrast, a high survival rate (99%) for unfrozen cells and a low rate (0.032%) for frozen cells were observed with *A. perforata* strain NIES-564 (Table 2; Additional file 1: Table S1, Fig. S3). Using mature colonies treated with 3% HA, > 100% survival was detected for unfrozen cells of *A. gubernaculifera*, compared to 0% for frozen colonies (Additional file 1: Table S1, Fig. S3). By contrast, mature colonies of *A. perforata* treated with 3% HA had the highest survival rate (5.5%) among frozen cell types and a moderate survival rate (57%) relative to the other unfrozen cell types (Table 2; Additional file 1: Table S1, Fig. S3). Therefore, the ability of HA and DMF to cross the cell membrane, and their toxic effects on cells in immature and mature colonies, differ between *A. gubernaculifera* and *A. perforata*.

In *A. gubernaculifera* strain NIES-4017, mature colonies treated with 3% DMF, 6% DMF and 3% HA exhibited 0% MPN survival after freezing and thawing. By contrast, immature colonies showed a < 0.2% MPN survival rate when treated with 3% DMF, 6% DMF, or 3% HA (Table 2). The difference in survival between immature and mature colonies of *A. gubernaculifera* could be attributed to differences in cell volume. Mature colonies of *Astrephomene* contain larger cells than immature colonies (Fig. 2A, B). Cell size is a critical factor for cryopreservation; cryopreserving large algal cells is problematic [26, 27]. However, in *A. perforata* strain NIES-564, mature colonies treated with 3% HA showed the highest MPN survival rate (5%) after freezing and thawing, while immature colonies treated with 3% HA had a 2.2% MPN survival rate (Table 2). Therefore, cell size may not critically influence the survival of *A. perforata* cells.

*A. gubernaculifera* strain NIES-4017 showed 11% MPN survival when immature colonies were treated with 6% DMF (Table 2). However, the four other strains of *A. gubernaculifera* showed < 0.1% MPN survival when immature colonies were treated with 6% DMF (Table 3). These *A. gubernaculifera* strains have been maintained by serial inoculations in liquid cultures since their establishment [16] (https://mcc.nies.go.jp/index_en.html). *A. gubernaculifera* strain NIES-4017 was originally established in 2014 from a single colony in a re-wetted soil sample [10], whereas other strains of this species were established from 1962 to 1981 (Table 1). During the cryopreservation of vegetative colonies of *Gonium pectorale*, 6% DMF as a cryoprotectant in two-step freezing was effective for cryopreservation, with MPN survival rates of ≥0.1% being maintained in 10 strains from the MCC-NIES [20]. However, three other strains of *G. pectorale* did not exhibit MPN survival rates ≥0.1% under identical cryogenic conditions (6% DMF) [20]. These three strains (NIES-2261, 469 and 570) have been maintained as growing subcultures since establishment of the original cultures in the period 1979–1994 [20]. Therefore, long-term maintenance of algal strains as growing subcultures by serial inoculation could decrease the survival rates of some strains of *Gonium* and *Astrephomene*.

## Conclusion

*A. gubernaculifera* colony maturation and cell volume are critical factors affecting survival after cryopreservation, possibly as a result of cryoprotectant permeability and/or toxicity (Additional file 1: Fig. S3). Large reproductive cells in mature colonies of *A. gubernaculifera* (Fig. 2B) do not survive 6% DMF treatment, which enables cryopreservation of small reproductive cells (Fig. 2A) (Table 2). Although this factor is not critical in *A. perforata* and may be species-specific (Table 2), the selection of cells of a suitable age or size may be important for successful cryopreservation in other colonial or multicellular volvocine genera.

Cryopreservation of some long-term-maintained strains of *A. gubernaculifera* (Table 3) and *G. pectorale* (NIES-2261, 469 and 570) is difficult [19]. However, strains established concomitantly are readily cryopreserved, particularly of complementary mating types of *G. pectorale* (NIES-2262, 468, and 569, respectively) [20]. Thus, during the long-term maintenance of cultures by subculturing, survival after cryopreservation may be decreased in certain strains of multicellular volvocine algae. Similarly, the inducibility of sexual reproduction and ability to perform normal morphogenesis gradually decrease during the long-term maintenance of cultures of multicellular volvocine species [10, 28]. Therefore, cryopreservation of newly established culture strains is important for future studies of multicellular volvocine algae.

The present study demonstrated that two species of *Astrephomene* can be cryopreserved using the optimal cryopreserved conditions for each species (Table 2). However, the survival rates are still low [11 ± 13% (0.36–33%) in *A. gubernaculifera* strain NIES-4017 and 5.5 ± 5.9% (0.12-12%) in *A. perforata* strain NIES-564 (Table 2)], which highlights that more effective conditions need to be standardized to obtain better survival.

## Supplementary Information

**Additional file 1: Table S1.** Comparison of effects of eight types of cryopreservation conditions (Table 2) on viabilities of *Astrephomene gubernaculifera* strain NIES-4017 (AG) and *A. perforata* strain NIES-564 (AP) without freezing and thawing, based on most probable number (MPN) methods. **Fig. S1.** Diagrammatic representation of the evolution of *Astrephomene* within the volvocine green algae, showing convergent evolution of germ-soma differentiation in spheroidal bodies. **Fig. S2.** Comparison of autotrophic (left, mVT medium) and photoheterotrophic (right, mVTAC medium) growth in six-day-old cultures of four multicellular volvocine species (*Astrephomene gubernaculifera* strain NIES-4017, *Volvulina steinii* strain NIES-4471, *Gonium pectorale* strain NIES-2863 and *Eudorina* sp. strain NIES-3984), based on the quantitative measurement (Additional file 2: Text S1, Table S2). **Fig. S3.** Comparison of mean rates of MPN survivability between two species of *Astrephomene* under 16 different conditions (Table 2; Additional file 1: Table S1).

**Additional file 2: Text S1.** Growth measurement of four multicellular volvocine species. **Table S2.** Composition of modified VT (mVT, for autotrophic growth condition) and modified VTAC (mVTAC, for photoheterotrophic growth condition) media.

## Data Availability

All data generated or analyzed during this study are included in this published article and its additional file.

## Abbreviations

CCA-UTEX: Culture Collection of Algae at the University of Texas at Austin
DMF: N,N-dimethylformamide
DMSO: Dimethyl sulfoxide
HA: Hydroxyacetone
MPN: The most probable number
MCC-NIES: Microbial Culture Collection at the National Institute for Environmental Studies
SAG: The Culture Collection of Algae at Goettingen University
VTAC: Volvox thiamin acetate
USVT: Urea soil *Volvox* thiamin

## References

[1] Kirk DL (1998). Volvox: molecular genetic origins of multicellularity and cellular differentiation.

[2] Umen J, Coelho S (2019). Algal sex determination and the evolution of anisogamy. Annu Rev Microbiol.

[3] Umen J, Herron MD (2021). Green algal models for multicellularity. Annu Rev Genet.

[4] Miller S, Nozaki H, Goodenough U (2022). Multicellular relatives of *Chlamydomonas*. The *Chlamydomonas* sourcebook, 3rd edition. Introduction to *Chlamydomonas* and its laboratory use.

[5] Nozaki H, Misawa K, Kajita T, Kato M, Nohara S, Watanabe MM (2000). Origin and evolution of the colonial Volvocales (Chlorophyceae) as inferred from multiple, chloroplast gene sequences. Mol Phylogenet Evol.

[6] Herron MD, Hackett JD, Aylward FO, Michod RE (2009). Triassic origin and early radiation of multicellular volvocine algae. Proc Natl Acad Sci U S A.

[7] Lindsey CR, Rosenzweig F, Herron MD (2022). Phylotranscriptomics points to multiple independent origins of multicellularity and cellular differentiation in the volvocine algae. BMC Biol.

[8] Yamashita S, Yamamoto K, Matsuzaki R, Suzuki S, Yamaguchi H, Hirooka S, Minakuchi Y, Miyagishima SY, Kawachi M, Toyoda A, Nozaki H (2021). Genome sequencing of the multicellular alga *Astrephomene* provides insights into convergent evolution of germ-soma differentiation. Sci Rep.

[9] Nozaki H (1983). Morphology and taxonomy of two species of *Astrephomene* (Chlorophyta, Volvocales) in Japan. J Jpn Bot.

[10] Yamashita S, Arakaki Y, Kawai-Toyooka H, Noga A, Hirono M, Nozaki H (2016). Alternative evolution of a spheroidal colony in volvocine algae: developmental analysis of embryogenesis in *Astrephomene* (Volvocales, Chlorophyta). BMC Evol Biol.

[11] Pocock MA (1954). Two multicellular motile green algae, *Volvulina* Playfair and *Astrephomene,* a new genus. Trans Roy Soc S Afr.

[12] Stein JR (1958). A morphological study of *Astrephomene gubernaculifera* and *Volvulina steinii*. Am J Bot.

[13] Brooks AE (1966). The sexual cycle and intercrossing in the genus *Astrephomene*. J Protozool.

[14] Brooks AE (1972). The physiology of *Astrephomene gubernaculifera*. J Protozool.

[15] Starr RC, Zeikus JA. UTEX—the culture collection of algae at the University of Texas at Austin. 1993 List of cultures. J Phycol. 1993(29):1–106. 10.1111/j.0022-3646.1993.00001.x.

[16] Kawachi M, Ishimoto M, Mori F, Yumoto K, Sato M, Noël M-H (2013). MCC-NIES. List of strains, 9th edition. Microalgae, endangered macroalgae and protists.

[17] Schlösser GG (1994). SAG - Sammlung von Algenkulturen at the University of Gottingen. Catalogue of strains 1994. Bot Acta.

[18] Mori F, Erata M, Watanabe MM (2002). Cryopreservation of cyanobacteria and green algae in the NIES-Collection. Microbiol Cult Coll.

[19] Nakazawa A, Nishii I (2012). Amidic and acetonic cryoprotectants improve cryopreservation of volvocine green algae. Cryo Lett.

[20] Nozaki H, Mori F, Tanaka Y, Matsuzaki R, Yamaguchi H, Kawachi M (2022). Cryopreservation of vegetative cells and zygotes of the multicellular volvocine green alga Gonium pectorale. BMC Microbial.

[21] Mori F (2007). Cryopreservation methods of microalgae. Microbiol Cult Coll.

[22] Fenwick C, Day JG (1992). Cryopreservation of *Tetraselmis suecica* cultured under different nutrients regimes. J Appl Phycol.

[23] Taylor R, Fletcher RL (1998). Cryopreservation of eukaryotic algae – a review of methodologies. J Appl Phycol.

[24] U.S. EPA (2013). Most probable number (MPN) calculator version 2.0 user and system installation and administration manual.

[25] APHA (2005). Standard methods for the examination of water and wastewater.

[26] Day JG (2007). Cryopreservation of microalgae and cyanobacteria. Methods Mol Biol.

[27] Whaley D, Damyar K, Witek RP, Mendoza A, Alexander M, Lakey JR (2021). Cryopreservation: an overview of principles and cell-specific considerations. Cell Transplant.

[28] Nozaki H (2008). Zygote germination in *Pleodorina starrii* (volvocaceae, chlorophyta). Biologia.

