# Supplementary material for: Cryopreservation of two species of the multicellular volvocine green algal genus Astrephomene
Source: BMC Microbiol. 2023 Jan 18;23:16. doi: 10.1186/s12866-023-02767-3 (PMC9847204; doi:10.1186/s12866-023-02767-3)
Supplement: Supplementary file 1 — Additional file 1: Table S1. Comparison of effects of eight types of cryopreservation conditions (Table 2) on viabilities of Astrephomene gubernaculifera strain NIES-4017 (AG) and A. perforata strain NIES-564 (AP) without freezing and thawing, based on most probable number (MPN) methods. Fig. S1. Diagrammatic representation of the evolution of Astrephomene within the volvocine green algae, showing convergent evolution of germ-soma differentiation in spheroidal bodies. Fig. S2. Comparison of autotrophic (left, mVT medium) and photoheterotrophic (right, mVTAC medium) growth in six-day-old cultures of four multicellular volvocine species (Astrephomene gubernaculifera strain NIES-4017, Volvulina steinii strain NIES-4471, Gonium pectorale strain NIES-2863 and Eudorina sp. strain NIES-3984), based on the quantitative measurement (Additional file 2: Text S1, Table S2). Fig. S3. Comparison of mean rates of MPN survivability between two species of Astrephomene under 16 different conditions (Table 2; Additional file 1: Table S1). [file 12866_2023_2767_MOESM1_ESM.pdf]

Table S1 Comparison of effects of eight types of cryopreservation conditions (Table 2) on viabilities of *Astrephomene gubernaculifera* strain NIES-4017 (AG) and *A. perforata* strain NIES-564 (AP) without freezing and thawing, based on most probable number (MPN) methods

Conditions			Total viability (range) %	MPN cell numbers in three cryotubes (/mL) (control)
Species /strain	Maturity of colonies/ cells	Cryo-protectant		
AG	Immature	3% DMF	77±75 (23-160)	21000, 42000, 150000 (92000)
AG	Immature	6% DMF	39±12 (25-46)	23000, 42000, 42000 (92000)
AG	Immature	3% HA	39±12 (25-46)	23000, 42000, 42000 (92000)

AG	Immature	6% HA	0.013±0.0072 (0.0067-0.021)	6.2, 9.4, 19 (92000)
AG	Mature	3% DMF	300±180 (180-500)	7400, 9200, 21000 (4200)
AG	Mature	6% DMF	140±69 (100-220)	4200, 4200, 9200 (4200)
AG	Mature	3% HA	210±150 (55-360)	2300, 9200, 15000 (4200)
AG	Mature	6% HA	0.071±0 (0.071-0.071)	3, 3, 3 (4200)
AP	Immature	3% DMF	74±45 (22-100)	9200, 42000, 42000 (42000)
AP	Immature	6% DMF	99±110 (22-220)	9200, 23000, 92000 (42000)

AP	Immature	3% HA	82±24 (55-110)	23000, 38000, 42000, (42000)
AP	Immature	6% HA	0±0 (0-0)	0, 0, 0 (42000)
AP	Mature	3% DMF	260±230 (22-500)	2300, 9200, 21000 (4200)
AP	Mature	6% DMF	120±85 (55-220)	2300, 4200, 9200 (4200)
AP	Mature	3% HA	57±40 (22-100)	920, 2100, 4200 (4200)
AP	Mature	6% HA	0±0 (0-0)	0, 0, 0 (4200)

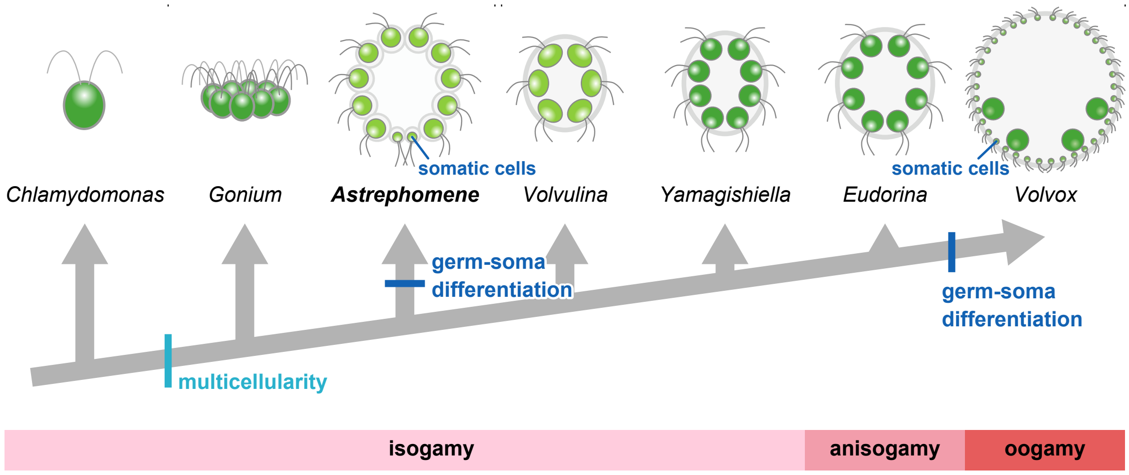

Fig. S1. Diagrammatic representation of the evolution of *Astrephomene* within the volvocine green algae, showing convergent evolution of germ-soma differentiation in spheroidal bodies.

The phylogeny is based on previous studies [1,2].

References

1. Lindsey CR, Rosenzweig F, Herron MD. Phylotranscriptomics points to multiple independent origins of multicellularity and cellular differentiation in the volvocine algae. BMC Biol. 2012;19:182. doi: 10.1186/s12915-021-01087-0.
2. Yamashita S, Arakaki Y, Kawai-Toyooka H, Noga A, Hirono M, Nozaki H. Alternative evolution of a spheroidal colony in volvocine algae: developmental analysis of embryogenesis in *Astrephomene* (Volvocales, Chlorophyta). BMC Evol Biol. 2016;16:243. doi: 10.1186/s12862-016-0794-x.

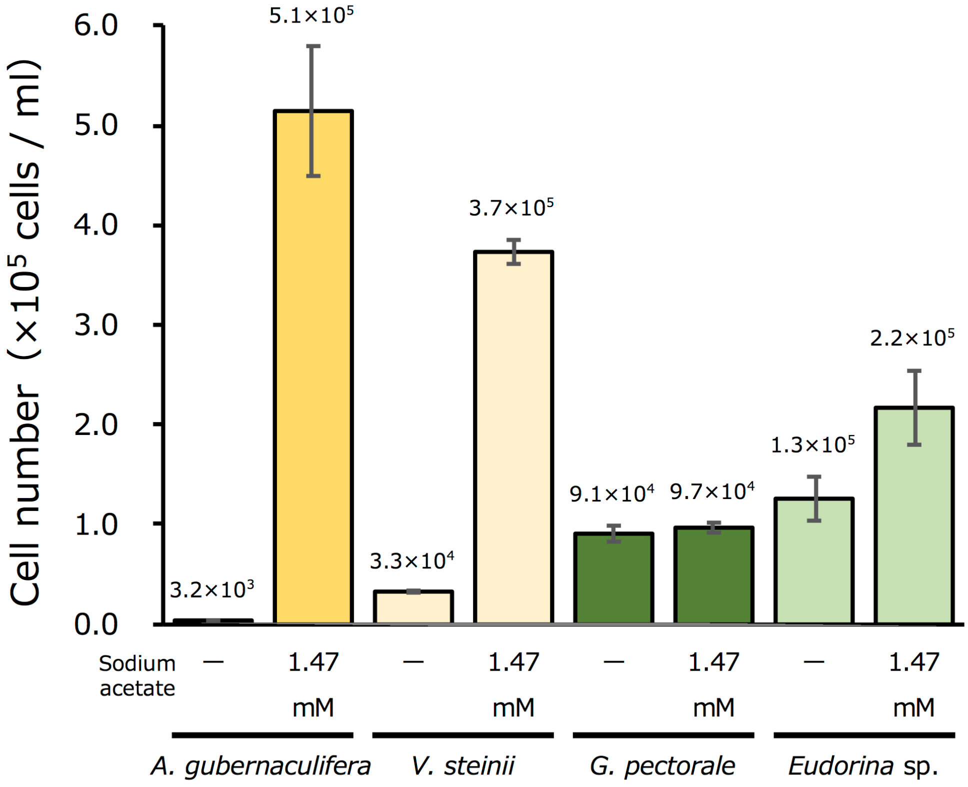

Fig. S2. Comparison of autotrophic (left, mVT medium) and photoheterotrophic (right, mVTAC medium) growth in six-day-old cultures of four multicellular volvocine species (*Astrephomene gubernaculifera* strain NIES-4017, *Volvulina steinii* strain NIES-4471, *Gonium pectorale* strain NIES-2863 and *Eudorina* sp. strain NIES-3984), based on the quantitative measurement (Additional file 2: Text S1, Table S2).

Data are represented as mean cell number (± SE, $n = 3$).

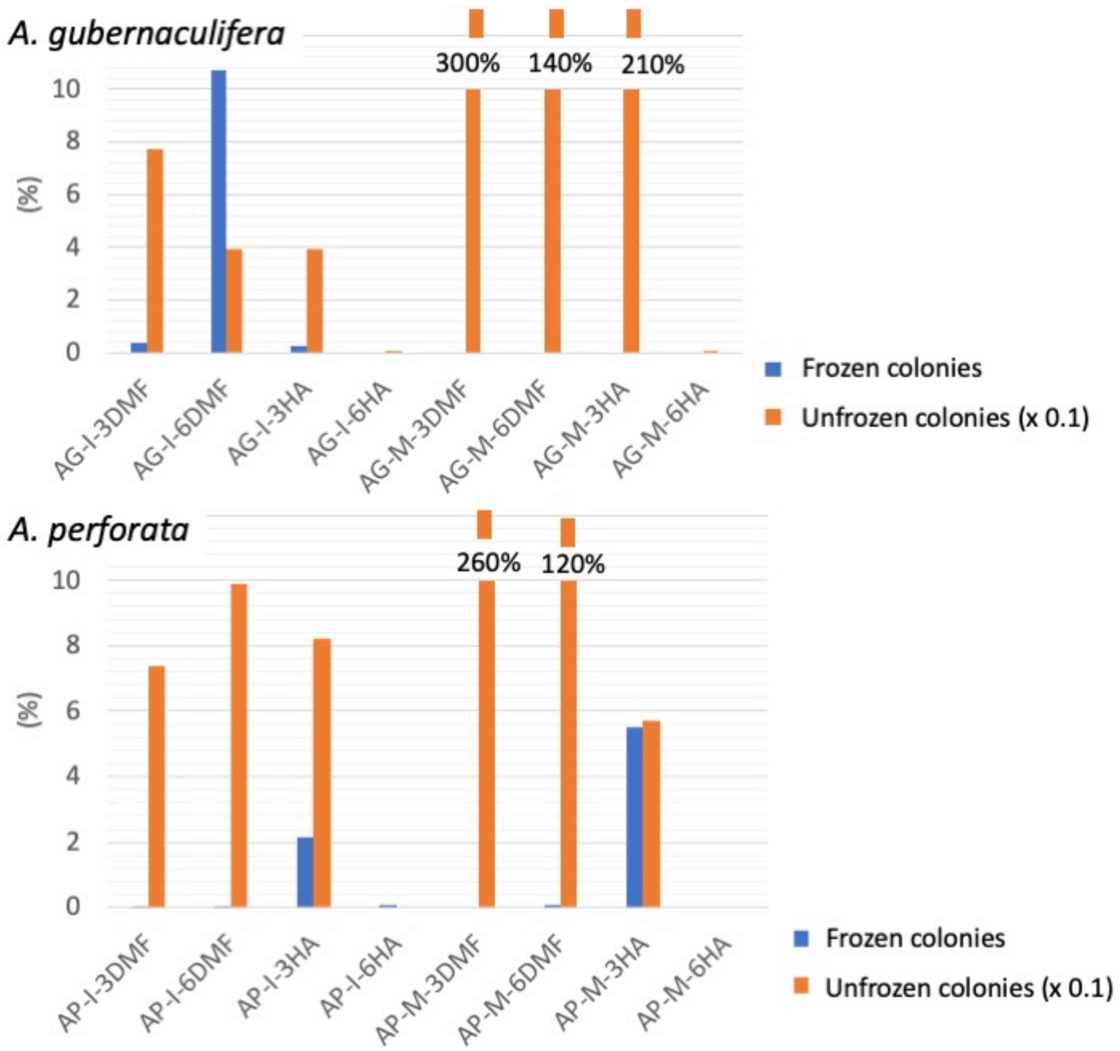

Fig. S3. Comparison of mean rates of MPN survivability between two species of *Astrephomene* under 16 different conditions (Table 2; Additional file 1: Table S1). AG: *A. gubernaculifera* strain NIES-4017. AP: *A. perforata* strain NIES-564. I: immature colonies (Fig. 2A, C) in asexual life cycle (Fig. 1A). M: mature colonies (Fig. 2B, D) in asexual life cycle (Fig. 1A). 3(6)DMP: 3(6)% N,N-dimethylformamide. 3(6)HA: 3(6)% hydroxyacetone.
