# Supplementary material for: Cryopreservation of two species of the multicellular volvocine green algal genus Astrephomene
Source: BMC Microbiol. 2023 Jan 18;23:16. doi: 10.1186/s12866-023-02767-3 (PMC9847204; doi:10.1186/s12866-023-02767-3)
Supplement: Supplementary file 2 — Additional file 2: Text S1. Growth measurement of four multicellular volvocine species. Table S2. Composition of modified VT (mVT, for autotrophic growth condition) and modified VTAC (mVTAC, for photoheterotrophic growth condition) media. [file 12866_2023_2767_MOESM2_ESM.pdf]

Text S1 Growth measurement of four multicellular volvocine species

Results

The results of our quantitative analyses of photoautotrophic growth and photoheterotrophic growth in the four colonial volvocine species are shown in Additional file 1: Fig. S2. Under autotrophic growth conditions, *Gonium pectorale* and *Eudorina* sp. exhibited 9.1×10^4 and 1.3×10^5 cells/ml in 6-day-old cultures, respectively. In contrast, *Astrephomene gubernaculifera* and *Volvulina steinii* showed only 3.2×10^3 and 3.3×10^4 cells/ml in 6-day-old cultures, respectively. When grown under photoheterotrophic conditions, growth of all four species increased. Although the growth of *A. gubernaculifera* and *V. steinii* under photoautotrophic conditions was extremely small when compared with that of the other two species, *A. gubernaculifera* and *V. steinii* grown under photoheterotrophic conditions showed extremely higher cell densities than those of two other species. These results are consistent with the previous reports [1,2] in that *A. gubernaculifera* and *V. steinii* grow abundantly in medium with acetate. In the present study, *A. gubernaculifera* showed the highest cell density (5.1×10^5 cells/ml) and the lowest cell density (3.2×10^3 cells/ml) under photoheterotrophic and photoautotrophic conditions, respectively, among the four (Additional file 1: Fig. S2).

Methods

A. gubernaculifera strain NIES-4017 and three other colonial volvocine species, *Volvulina steinii* NIES-4471, *Gonium pectorale* strain NIES-2863 and *Eudorina* sp. strain NIES-3984, were obtained from the Microbial Culture Collection at the National Institute for Environmental Studies (NIES-Collection: https://mcc.nies.go.jp/index_en.html; [22].The cultures of the four species were maintained in 10 ml modified VT medium (mVT, lacking major organic compounds) for "photoautotrophic growth" or modified VTAC medium (mVTAC) (containing 200 mg/L sodium acetate・$3H_2O$) for "photoheterotrophic growth" (Additional file 2: Table S2), at 25.5°C under continuous light (130-170 µmol m^{-2} s^{-1}).

In order to examine the effects of acetate on growth, two different media, mVT (without sodium acetate・$3H_2O$) and mVTAC (mVT with 200 mg/L sodium acetate・

$3H_2O$; Additional file 1: Table S2) were used for growth measurements of each strain. Two- to six-old cultures of the four strains (*A. gubernaculifera* strain NIES-4017, *V. steinii* strain NIES-4471, *G. pectorale* strain NIES-2863 and *Eudorina* sp. NIES-3984) in mVT or mVTAC were used for pre-culture for inoculation. To measure the cell density of pre-cultures, 1 mL of each culture was transferred to a new tube, from which 300 µL culture was picked up and fixed with 30 µL 25% glutaraldehyde. Then 20 µL of the well-suspended fixed sample were put on a slide with Vaseline (for assuring the height) and covered by a coverslip. The cell number of the preparation was counted under BX-53 and BX-60 microscopes (Olympus, Tokyo, Japan). The counts are conducted based on three different preparations for each culture. Based on the cell density calculated as described above, the pre-culture containing 1.0×10^3 cells was inoculated into a new medium (10 mL) and cultured at 25.5°C under continuous light (130-170 µmol m^{-2} s^{-1}). After 6 days, the cell density of each culture was examined three times as described above. Experiments were carried out with three replicates for each culture.

References

1. Carefoot, J. R. (1967) Nutrition of *Volvulina* Playfair. *J. Protozool.* 14:15-18 doi: 10.1111/j.1550-7408.1967.tb01439.x.
2. Brooks, A. E. (1972) The physiology of *Astrephomene gubernaculifera*. *J. Protozool.* 19:195-199 doi: 10.1111/j.1550-7408.1972.tb03435.x.

Table S2 Composition of modified VT (mVT, for autotrophic growth condition) and modified VTAC (mVTAC, for photoheterotrophic growth condition) media

mVT medium [a]

Components	/ 1 L	Final concentrations
$Ca(NO_3)_2 \cdot 4H_2O$	118 mg	0.50 mM
$NaH_2PO_4 \cdot 2H_2O$	9 mg	0.06 mM
$Na_2HPO_4 \cdot 12H_2O$	36 mg	0.10 mM
$MgSO_4 \cdot 7H_2O$	40 mg	0.16 mM
KCl	50 mg	0.67 mM
Vitamin B_{12}	0.1 µg	0.07 nM
Biotin	0.1 µg	0.41 nM
Thiamine HCl	10 µg	29.6 nM
P IV metals (below)	3 mL	
Distilled water	to 1 L	
pH 7.6		

mVTAC medium [b]

1 L mVT medium

200 mg sodium acetate $\cdot 3H_2O$ (the final concentration is 1.47 mM)

P IV metals stock solution [a]

Components	/ 1 L	Final concentrations in mVT (AC)
$Na_2EDTA \cdot 2H_2O$	1000 mg	8.06 µM
$FeCl_3 \cdot 6H_2O$	196 mg	2.18 µM
$MnCl_2 \cdot 4H_2O$	36 mg	0.55 µM
$ZnCl_2$	10 mg	0.23 µM
$CoCl_2 \cdot 6H_2O$	4 mg	0.05 µM
$Na_2MoO_4 \cdot 2H_2O$	5 mg	0.03 µM

[a] Based on Provasoli & Pintner [1].

[b] Based on Nozaki *et al.* [2].

References

1. Provasoli L, Pintner IJ. Artificial media for fresh-water algae: problems and suggestions. In Tryon CAJr, Hartmann RT, editors. The Ecology of Algae. Spec. Pub. No. 2: Pymatuning Laboratory of Field Biology, University of Pittsburgh: Pittsburgh; 1960, p. 84-06.
2. Nozaki H, Kuroiwa H, Mita T, Kuroiwa T. *Pleodorina japonica* sp. nov. (Volvocales, Chlorophyta) with bacteria-like endosymbionts. Phycologia 1989;28:252-67 doi: 10.2216/i0031-8884-28-2-252.1.
